# Bone Grafting with Albumin-Impregnated Bone Allograft After Odontogenic Cyst Removal

**DOI:** 10.3390/jcm14124173

**Published:** 2025-06-12

**Authors:** Anna Rangics, Gábor Dénes Répássy, Dóra Hargas, Csaba Dobó-Nagy, Szabolcs Gyulai-Gaál, András Molnár, László Simonffy

**Affiliations:** 1 Department of Dentoalveolar Surgery, Dental Training Centre, Faculty of Dentistry, Semmelweis University, 1085 Budapest, Hungary; rangics.anna@semmelweis.hu (A.R.); gyulai-gaal.szabolcs@semmelweis.hu (S.G.-G.); simonffy.laszlo@semmelweis.hu (L.S.); 2 Department of Otorhinolaryngology, Head and Neck Surgery, Semmelweis University, Szigony u. 36, 1083 Budapest, Hungary; 3 Faculty of Medicine, Semmelweis University, Üllői út 26, 1085 Budapest, Hungary; hargasdora@gmail.com; 4 Department of Oral Diagnostics, Faculty of Dentistry, Semmelweis University, 1085 Budapest, Hungary; dobo-nagy.csaba@semmelweis.hu; 5 Opera Clinic, Protone Audio Kft, Lázár u. 4, 1065 Budapest, Hungary; andrasm94@gmail.com

**Keywords:** odontogenic cyst, bone-albumin, bone grafting, cyst removal, tissue regeneration

## Abstract

**Background:** Odontogenic cysts can damage the surrounding bone tissue as they grow, making it essential to implement effective regenerative strategies tailored to each patient. Personalised approaches in oral surgery, such as selecting the most suitable bone graft materials, can lead to improved treatment outcomes. Filling the bone defect created after cyst removal, root resection, or extraction with a bone graft material can stabilise the weakened tooth and promote faster bone regeneration. This article shares our experiences with the therapeutic effects of albumin-coated bone allograft (BoneAlbumin^®^) placed in the bone defect following cyst removal in the oral cavity, compared to cases where the defect was left untreated (controls). **Methods:** The study involved thirty patients who underwent the removal of maxillary odontogenic cysts. In 15 of these patients, the bone defect was filled with albumin-coated bone allograft (BoneAlbumin^®^, OrthoSera, Budapest, Hungary). In the control group, which consisted of 15 patients, the defect was left untreated. A consistent surgical protocol was adhered to throughout the study. Follow-up periapical X-rays were taken immediately after surgery as well as at 6 and 12 weeks post-surgery, using a standardised template. These images were used to assess the shrinkage and healing of the defect caused by the cyst. Measurements were adjusted to reference points to account for potential distortions in the X-rays. **Results:** The control and study groups exhibited no statistically significant differences in their basic parameters. Additionally, there was no notable difference in the sizes of postoperative defects between the two groups. However, statistical analysis revealed a significant difference in the changes in defect size (∆defect size) between the groups at both 6 weeks (*p* < 0.000001) and 12 weeks (*p* = 0.000296). This suggests that the BoneAlbumin^®^-graft group experienced significantly greater changes in defect size over time. **Conclusions:** The use of BoneAlbumin^®^ graft leads to a markedly better reduction in defect size as time progresses, although these changes have only been compared to graft-free healing.

## 1. Introduction

A cyst is a pathological cavity lined with epithelium and filled with fluid but not pus. Odontogenic cysts are specific conditions that affect the tissues in the oral and maxillofacial regions. These cysts develop due to inflammatory or developmental factors related to the epithelium of the tooth-forming apparatus. As these cysts grow, they increase hydrostatic pressure in the affected area, leading to the atrophy of surrounding bone tissues. As a result, the roots of nearby teeth may become displaced. Intrabony cysts often do not cause any symptoms, and clinical issues typically arise only due to their size or potential for inflammation. These cysts are most commonly discovered incidentally during radiological examinations [1].

Periapical cysts, also referred to as radicular or periradicular cysts, represent the most prevalent type of odontogenic cysts, accounting for up to 60% of all cases. These cysts typically arise due to chronic inflammation resulting from the necrosis of the dental pulp, often stemming from untreated caries or dental trauma. They originate from the epithelial rests of Malassez, which become activated by the inflammatory process. Radiographic examination usually reveals a well-defined radiolucency at the apex of a non-vital tooth. Anatomically, these cysts are most frequently found in the anterior region of the maxilla and around the premolars in the mandible. This distribution may be attributed to the increased susceptibility of these areas to dental injuries and carious lesions [2].

There are several surgical approaches for treating odontogenic cysts. Two common initial methods are decompression and marsupialisation, both of which are aimed at reducing the cyst’s volume and preventing it from affecting nearby anatomical structures. In the decompression technique, a surgical stent is sutured to create a connection between the cystic cavity and the oral mucosa. On the other hand, marsupialisation involves making an incision through the lesion. This incision is then stitched to the fibrous wall of the cyst and the adjacent oral mucosa, establishing a connection between the cystic cavity and the oral cavity [3]. Another approach is primary cyst removal, which entails the immediate excision of the cyst. After the cyst is removed, the resulting cavity may be left untreated or filled with bone material. This material can be either autologous (from the patient’s own body) or a bone substitute. Regardless of the surgical method used or the type of filling material chosen, the key factor is effectively restoring bone within the defect [4].

Primary cyst removal is a common dento-alveolar surgical procedure. Numerous studies have investigated bone grafting techniques for repairing bone defects that arise from this procedure. Research has shown that natural bone regeneration occurs in a supportive environment with adequate blood supply and mesenchymal cells. However, in the case of ‘critical-sized defects’, complete healing cannot happen without mechanical support [5]. For such defects, external materials are necessary to facilitate bone regeneration [6]. There is currently no standardised definition for what constitutes a ‘critical-sized defect.’ Generally, it refers to a defect that cannot heal on its own, even with surgical stabilisation, and requires additional surgical intervention, such as autologous bone grafting. The literature suggests that a defect is typically considered significant if its length exceeds 1 to 2 cm and if there is more than a 50% loss in the circumference of the bone [5,7,8].

The growing interest in various bone graft materials is largely driven by the increasing demand for aesthetic outcomes and the rising popularity of implant restorations. This trend has prompted a substantial increase in scientific research focused on these materials, resulting in a wider range of available bone graft options. Consequently, progress is being made toward identifying the ideal bone graft material. However, it is crucial to conduct in vivo examinations alongside in vitro studies to fully understand the efficacy of these materials. In recent years, numerous studies have been published on serum albumin-impregnated bone allografts. One such product, BoneAlbumin^®^—to the authors’ best knowledge, the only commercially available albumin-coated bone allograft—has been successfully used in orthopaedic procedures, alveolar ridge preservation, and maxillary sinus elevation. This suggests that it may also be beneficial for treating bony defects following the removal of dentoalveolar cysts [9,10,11,12,13].

Allografts are sourced from another individual of the same species and are subjected to sterilisation, processing, and preservation in bone banks. They provide several advantages, including osteoconductivity, an ideal human tissue structure, excellent biomechanical properties, volume stability, and the ability to reconstruct extensive defects without causing morbidity at the donor site [9,11]. However, during the production of bone grafts, the proteins can denature, which results in minimal osteoinductivity. This reduced effectiveness in promoting bone growth can lead to slower bone remodelling and an increased likelihood of being absorbed by the body [14,15].

Given all the biomedical benefits of albumin and the bone regeneration advantages of albumin-impregnated bone grafts, this study aims to compare the therapeutic effects of placing BoneAlbumin^®^ in the bone cavity after the removal of odontogenic cysts with leaving the defect empty (control group).

## 2. Materials and Methods

### 2.1. Patient Selection

Thirty adult patients diagnosed with an odontogenic cystic lesion were referred to the Department of Dentoalveolar Surgery at the Faculty of Dentistry, Semmelweis University, Budapest, Hungary. The inclusion criteria for the study required clinical and radiological signs of a radicular cyst, such as a necrotic pulp in the affected teeth and the presence of a cyst developing at the root apex. Additionally, all patients provided consent to participate in the study. The exclusion criteria comprised the following: previous radiation treatment in the head and neck area, the use of bisphosphonates or other antiresorptive medications, and chronic diseases affecting the bones, metabolism, blood coagulation, cardiovascular system, immune system, or severe cancer-related conditions. Patients with untreated illnesses, those who could not provide consent or cooperate, individuals with psychological conditions, and pregnant women were also excluded. All patients were thoroughly informed about the procedures and signed consent forms. Preoperative planning utilised previously taken X-rays. The study adhered to the guidelines of the Declaration of Helsinki and received approval from the Institutional Ethics Committee of Semmelweis University (protocol code: 31068–7/2018/EÜIG, approval date: 6 September 2018). Each patient underwent both clinical and radiological examinations, which included an orthopantomogram and a periapical radiograph. Following the clinical diagnosis, the patients were randomly assigned to one of two groups. Patients in the first group (*n* = 15) received BoneAlbumin^®^ after cyst removal, while the second group (control group, *n* = 15) did not receive any bone graft material, leaving the bone defects empty. The key details regarding the study population are provided in Table 1.

### 2.2. Production of the Bone Grafting Material

BoneAlbumin^®^ is created through a meticulous process that begins with the collection of bone from femoral heads obtained during hip replacement surgeries, conducted under the operational licence of the West-Hungarian Tissue Bank. The harvested bone is processed to produce autolysed, antigen-extracted allogenic bone, following the method developed by Urist. The preservation process involves freeze-drying the bone under aseptic conditions, followed by sterilisation with ethanol (EtOH). After the initial freeze-drying, the bone grafts are immersed for one minute in a sterile 10% human serum albumin solution, using low-salt-content Biotest human albumin infusion (Biotest Pharma GmbH, Dreieich, Germany). A second freeze-drying is then conducted under the same parameters to finalise the production of BoneAlbumin^®^ [11].

BoneAlbumin^®^ is a unique bone allograft infused with human serum albumin that aims to overcome the limitations of traditional allografts [9,11,12,13,16,17].

### 2.3. Molecular Background of Albumin Coating

Albumin is one of the most abundant proteins found in plasma and plays several essential metabolic roles. These roles include regulating oncotic pressure, binding to and transporting various molecules, scavenging free radicals, and modulating immune responses as well as blood coagulation [18]. Human serum albumin (HSA) has a molecular weight ranging from 66.5 to 69 kDa and is composed of 585 amino acids [19]. This non-glycosylated polypeptide has a globular, heart-like shape and consists of six helical subdomains formed by three homologous domains (I, II, and III). Each of these domains contains two subdomains that share similar structural features [19,20].

HSA exhibits high bioactivity and solubility, making it an excellent choice for a temporary coating material that improves the biocompatibility of the coated biomaterial [21]. There are various techniques for immobilising albumin on the surface of biomaterials; however, simple lyophilisation is adequate for coating a bone allograft [22].

Numerous studies have demonstrated that albumin coatings improve materials’ bio- and immune compatibility, tissue formation, enhance corrosion resistance, and provide antibacterial and anticoagulant properties [16,17,23,24,25,26]. HSA exhibits robust cell adhesive properties in more physiological scaffolds, including human bone allografts [27,28,29].

Albumin precoating creates a thin protein layer that enhances surface hydrophilicity and minimises the biological response of hydrophobic materials when they come into contact with blood. This process is known as ‘albumin passivation’ [26,30]. Research indicates that materials coated with native albumin can reduce both the number of platelets adhering to the surface and their levels of activation. However, when the structure of albumin is modified through crosslinking, platelets can fully adhere to and activate on the altered albumin layer [22]. Various commercial products use albumin coatings, including perfusion systems, catheters, cannulas, and extracorporeal life support systems [11,16,31,32,33,34,35].

### 2.4. Surgical Protocol

**Figure 1:**
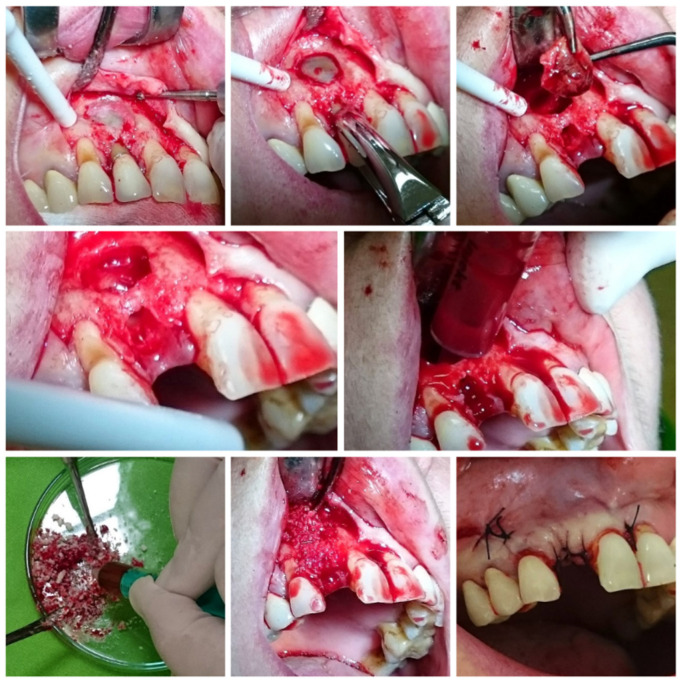
Cyst removal, followed by filling the bone defect with BoneAlbumin^®^.

All patients underwent the same surgical protocol for cyst removal, performed by a single dento-alveolar surgeon. Before the surgery, the patients were instructed to rinse their mouths with 0.2% chlorhexidine mouthwash for one minute. During the procedure, a full-thickness flap was elevated under local anaesthesia. A bone window was created using ball-shaped steel surgical burs. Once the cyst was removed, BoneAlbumin^®^ was packed into the defect with light pressure. In the control group, no bone graft material was used after cyst removal. BoneAlbumin^®^ is hygroscopic and forms a mouldable paste when mixed with blood or physiological saline. After completing these steps, primary closure of the flap was performed, with the margins stabilised using single interrupted sutures (see Figure 1). The cyst that was removed was examined during the surgery and confirmed to be a radicular cyst.

Postoperatively, antibiotics (875 mg of amoxicillin and 125 mg of clavulanate, to be taken twice a day for seven days, or if there are side effects or known allergy to penicillin, 300 mg clindamycin four times a day for four days), anti-inflammatory drugs (50 mg diclofenac, to be taken three times a day for three days), and chlorhexidine mouth-wash (to be used twice a day for two weeks from the day after surgery) were prescribed. The sutures were removed after ten days.

### 2.5. Follow-Up

The maximum linear width of the cyst defect was measured three times: immediately after surgery and at 6 and 12 weeks postoperatively using periapical radiographs.

**Figure 2:**
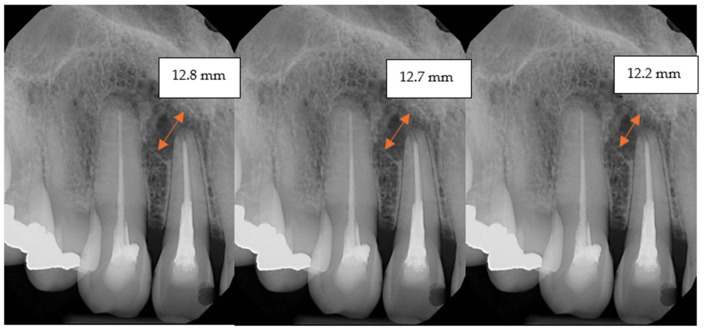
A series of periapical radiographs from the control group, taken at baseline, as well as at 6 weeks and 12 weeks. To enhance the visibility of defect sizes, the diameters are shown in millimetres in each figure.

The defect measurements were manually taken by two independent researchers using periapical radiographs (Figure 2), with each measurement repeated three times to ensure reproducibility.

**Figure 3:**
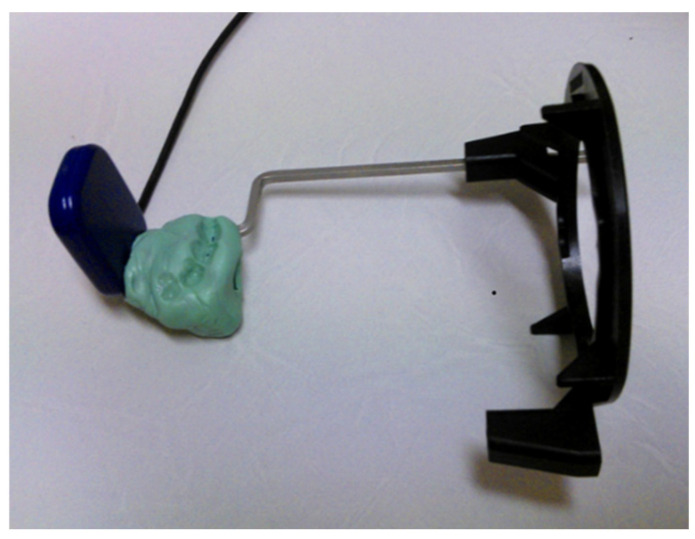
Silicone template for the intraoral X-ray.

To minimise variability, a custom-made silicone positioning template was used (see Figure 3) for standardising radiographs. This ensured consistent angulation and depth at each follow-up appointment. Radicular cysts typically appear as round, unilocular, lucent lesions in the periapical region. Additionally, obtaining CBCT images subjects the patient to higher radiation doses, which are not routinely recommended for apical lesions and are not widely available in primary care dentistry.

**Figure 4:**
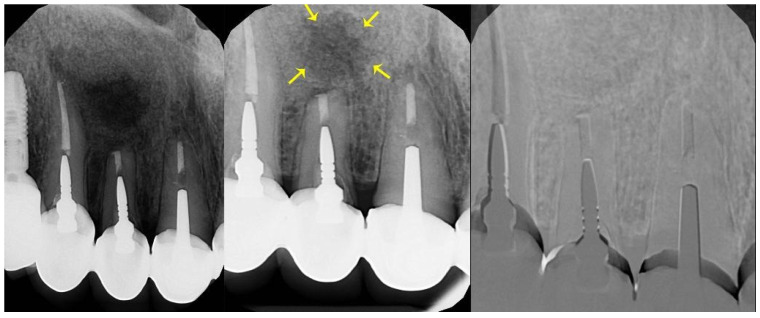
Baseline, follow-up (after 12 weeks), and subtracted radiographs of an upper lateral incisor. Yellow arrows point to newly formed trabeculae oriented towards the centre of the lesion.

After taking measurements, the baseline and follow-up radiographs were used to create a digital subtraction radiographic image (see Figure 4). Following the augmentation, the grafted area appears radio-opaque, making it difficult to visualise the borders directly. To tackle this challenge, the assessment of defect shrinkage was not solely based on linear opacity differences; the reappearance of trabecular bone patterns within the previously defected area was also considered. This is a recognised indicator of bone healing in periapical radiography.

Additionally, digital subtraction radiography (DSR) was used, which highlights changes in bone density over time by subtracting baseline images from follow-ups. This method emphasises the presence of newly formed trabeculae, even when exact borders are obscured.

No participants were lost to follow-up, and all scheduled data points (baseline, 6 weeks, and 12 weeks) were collected for each subject.

### 2.6. Statistical Analysis

Statistical analysis was conducted using IBM SPSS version 25 (IBM Corporation, Armonk, NY, USA). The normality of the data was assessed using the Shapiro–Wilk test, which confirmed that both groups followed a normal distribution. As a result, continuous variables were presented as means along with their standard deviations (SDs). To identify any statistically significant differences, a two-sample *t*-test was employed. Additionally, the chi-squared test was used for categorical data analysis. The significance level was set at *p* < 0.05.

## 3. Results

Table 1 provides basic information about the study population.

Table 1 indicates that there were no statistically significant differences in age (*p* = 0.37, *t*-value: –0.32) or sex (*p* = 0.46) between the two groups. This suggests that the groups are suitable for further analysis. Additionally, the occurrence of complications, specifically fistula formation, did not differ significantly between the two groups (*p* = 0.67).

**Figure 5:**
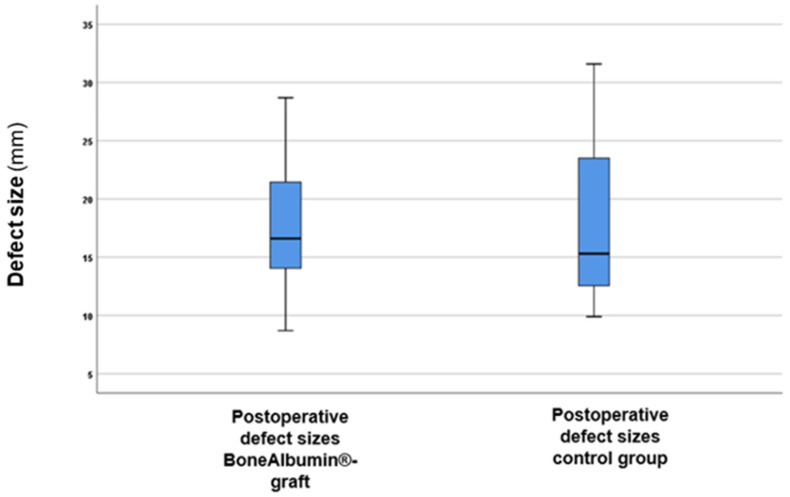
Postoperative defect sizes between the BoneAlbumin^®^-graft group and the control group. The boxes represent the middle 50% of the data, while the whiskers indicate the upper and lower 25%. The black line in each box indicates the median values: 16.6 mm for the BoneAlbumin^®^-graft group and 15.3 mm for the control group.

In the initial stage of the analysis, the postoperative defect sizes between the BoneAlbumin^®^ graft and control groups were compared. The results are displayed in Figure 5.

As shown in Figure 5, there were no significant differences in defect sizes between the two groups. A statistical analysis using the *t*-test revealed that the differences were not statistically significant (*p* = 0.23, *t*-value: 0.736). This suggests that the defect sizes were comparable between the groups.

The next phase of the investigation involved analysing the changes in defect sizes over time.

**Figure 6:**
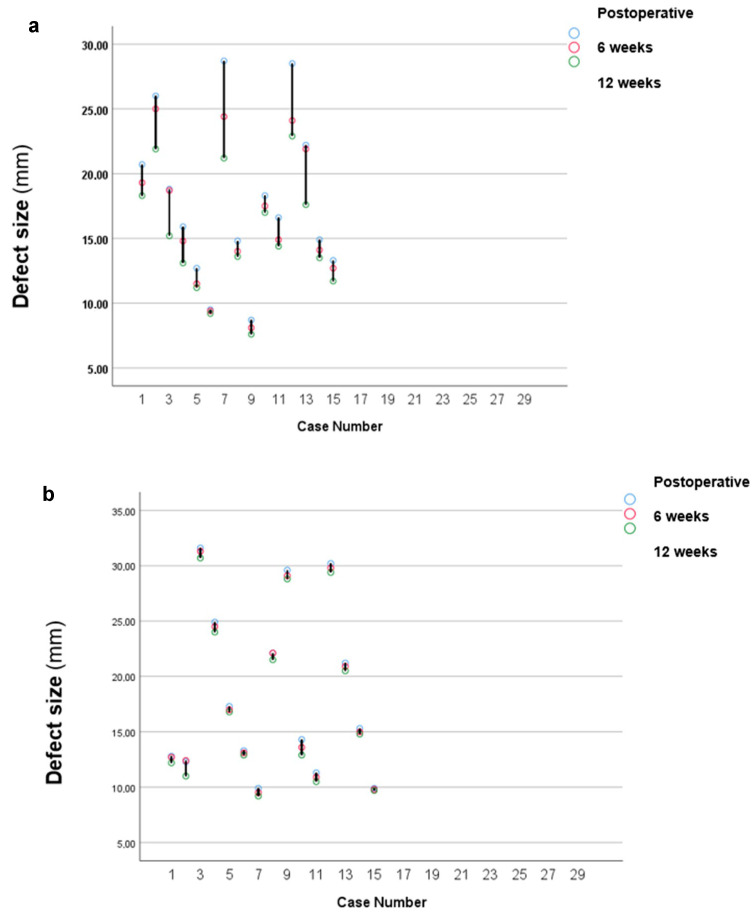
The change in defect sizes over time was measured during the 6-week and 12-week follow-ups for both groups: (**a**) the BoneAlbumin^®^-graft group and (**b**) the control group. The blue circles represent the postoperative values, the red circles indicate the values measured at 6 weeks, and the green circles show the values recorded at 12 weeks.

**Figure 7:**
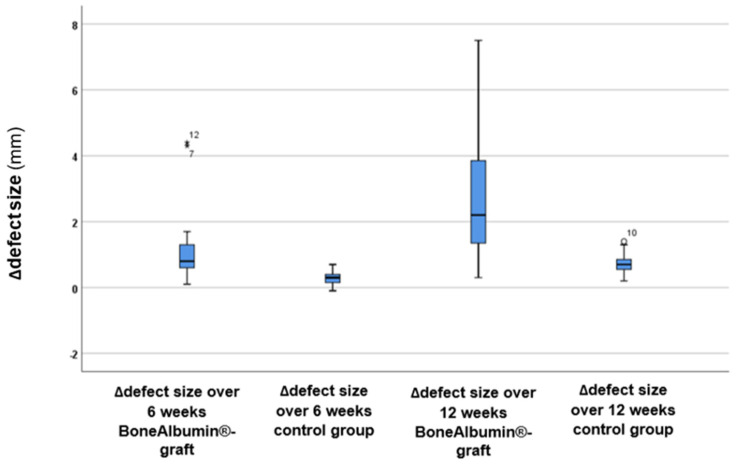
∆defect size values between the two groups at the 6-week and 12-week follow-ups. The boxes represent the middle 50% of the data, while the whiskers indicate the upper and lower 25%. The black line within each box indicates the median values in the BoneAlbumin^®^ graft and control groups: 14.9 mm vs. 15 mm after 6 weeks, and 14.4 mm vs. 14.8 mm after 12 weeks. The circles, asterisks, and numbers indicate the outliers (i.e., a 4.3 mm change in the ∆defect size over 6 weeks using BoneAlbumin^®^ grafts and a 1.4 mm change in the control group after 12 weeks of follow-up).

As shown in Figure 6, there were significant differences in defect size changes between the two groups, with the BoneAlbumin^®^-graft group exhibiting greater changes. Accordingly, the ∆defect size values were calculated (Figure 7). Statistical analysis using the *t*-test revealed a statistically significant difference in the ∆defect size values between the two groups at both 6 weeks (*p* < 0.000001*, *t*-value: –5.75) and 12 weeks (*p* = 0.000296*, *t*-value: 3.87). These results indicate that the BoneAlbumin^®^-graft group experienced significantly greater changes in defect size over time (Table 2). Thus, the use of BoneAlbumin^®^ graft leads to a markedly better reduction in defect size as time progresses.

## 4. Discussion

This study aimed to analyse the effects of BoneAlbumin^®^ grafts on reducing defect sizes following the removal of radicular cysts. The investigation found that the use of BoneAlbumin^®^ grafts resulted in significantly greater reductions in defect sizes compared to the control group, which did not use any grafts. The limitations of traditional autografts and allografts underscore the need for alternative materials. Albumin, a widely available protein known for its significant regenerative effects, can be produced at a low cost, making it an excellent choice for bone replacement. When combined with bone substitute materials, albumin enhances their osteoinductive capacity, reduces resorption, lowers the risk of infection due to its antimicrobial properties, and accelerates the healing process [36].

In addition to their anticoagulant properties, albumin coatings can help prevent bacteria from adhering to biomaterial surfaces. Previous studies have demonstrated that impregnating solid substrates with albumin can reduce the adhesion of *Staphylococcus aureus* [37]. For instance, Cometta et al. showed that physically immobilising 1% and 5% human serum albumin (HSA) on the surface of 3D printed medical-grade polycaprolactone (mPCL) scaffolds decreased colonisation by *S. aureus*. When these scaffolds were treated with HSA and then crosslinked or stabilised using tannic acid, the resulting coatings were uniform, stable, and displayed enhanced antibacterial properties [38]. Additionally, a study conducted by An et al. explored the effect of albumin coating on titanium implants to prevent infections in rabbits. All implants were exposed to a suspension of *Staphylococcus epidermidis* before implantation. The results indicated a significant reduction in the infection rate: from 62% for rabbits with non-coated implants to just 27% for those with albumin-coated implants [39].

Although albumin typically inhibits cell adhesion, research has shown that albumin coatings can actually promote cell growth, enhance tissue integration, and improve biocompatibility in various biomedical applications [16,17,25,27,28,29]. Studies indicate that freeze-dried albumin coatings are more effective than other coatings for promoting cell attachment and proliferation on bone grafts, particularly in human bone. This improvement accelerates healing and reduces recovery time for critical bone defects [16,35]. It is hypothesised that an increased concentration of local albumin has a recruiting effect on mesenchymal stem cells, encouraging their migration from the bone marrow or bloodstream. Albumin is absorbed during the healing process, and its presence increases the number of stem cells involved in bone regeneration, which can differentiate into bone-forming cells [16,23]. Clinical studies indicate that using BoneAlbumin^®^ leads to lower postoperative pain and quicker integration compared to uncoated grafts [12]. Additionally, albumin coatings have immunomodulatory effects that help diminish inflammatory responses and promote healing [32]. Beyond their application in bone grafts, albumin-coated materials show promise in soft tissue regeneration and enhance the corrosion resistance of implantable biomaterials. This makes albumin an essential coating for various medical applications [39].

**Figure 8:**
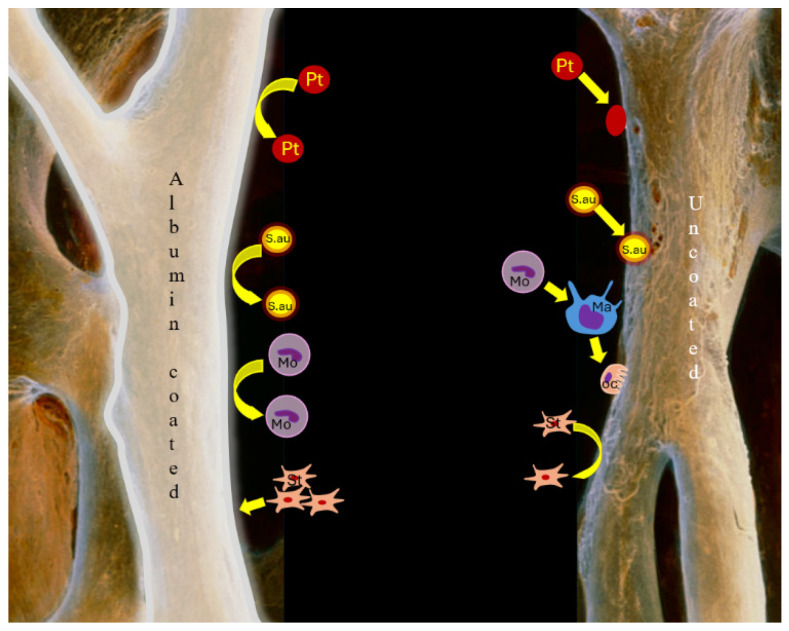
Differences between the albumin-coated and uncoated bone graft materials (abbreviations: Ma = Macrophages; Mo = Monocyte; Pt = Platelet; S.au = *S. aureus*, St = Stem cells, Oc = Osteoclast). The albumin-coated graft material significantly enhances bone regeneration by promoting stem cell recruitment, reducing bacterial adhesion, and creating a favourable environment for osteogenesis. In contrast, uncoated materials are less effective in these areas and carry a higher risk of complications such as infection and inflammation. This figure was created by the authors themselves.

Figure 8 illustrates the differences between coated and uncoated bone graft materials. The albumin coating on the bone grafts enhances the recruitment of mesenchymal stem cells from nearby sources, such as blood or bone marrow. These stem cells are directed toward the coated surface (as indicated by the yellow arrows), where they can differentiate into bone-forming cells. The albumin layer serves as a barrier, reducing the adhesion of *S. aureus* and thereby lowering the risk of infection. Additionally, platelets adhere to the albumin surface and initiate a regenerative environment by releasing growth factors that support stem cell activity and tissue healing. Monocytes and macrophages are also drawn to the coated surface, promoting a balanced immune response and aiding in regeneration. This environment encourages the differentiation of stem cells into osteoblasts rather than osteoclasts, ultimately enhancing bone formation.

In the case of uncoated bone grafts that lack an albumin coating, there is minimal recruitment of stem cells, which reduces their regenerative potential. Without the albumin, *S. aureus* can adhere more easily to the surface, increasing the risk of infection. Additionally, platelet adhesion and activation are less pronounced, leading to a decreased release of essential growth factors necessary for healing. Monocytes and macrophages may respond more aggressively, potentially causing inflammation instead of facilitating healing. Furthermore, the uncoated surface may promote osteoclast activity, resulting in bone resorption rather than the formation of new bone.

Recent scientific research has made bone allografts with various impregnations more accessible for everyday use, and methods for their production have improved significantly. However, this technique may result in a loss of the allografts’ bone-forming properties. During the harvesting process, the host’s immunogenic activity is eliminated, causing the living osteogenic cells on the surface of the allograft to die and the osteoinductive proteins to denature. To restore the osteoinductive surface, grafts can be impregnated with proteins such as BoneAlbumin^®^, which can rejuvenate their bone-forming characteristics and promote faster integration and remodelling while minimising the risk of resorption. Additionally, the albumin coating provides antibacterial properties, which is particularly important in oral surgery [40,41,42].

In the group where the cystic cavity was filled with BoneAlbumin^®^, we observed a significantly faster rate of bone regeneration. This leads us to conclude that BoneAlbumin^®^ could be an effective material for bone substitution.

### Limitations

This study has several limitations that should be acknowledged. First, the sample size was restricted to 30 patients, which may limit the statistical power and generalisability of the findings. However, the study was designed as a pilot investigation to assess the early clinical efficacy of BoneAlbumin^®^ in a specific surgical context and serves as a foundation for future, larger-scale studies. Second, the follow-up period was limited to 12 weeks, as this timeframe was selected to capture the most active phase of early bone remodelling. While this duration is consistent with other studies on early-phase bone regeneration, it does not allow for the assessment of long-term outcomes, such as complete bone maturation or the stability of grafts over time. Furthermore, the study utilised two-dimensional periapical radiographs to measure defects. Although this method was standardised with custom silicone positioning templates and enhanced by digital subtraction radiography, it may not accurately capture volumetric changes as effectively as three-dimensional imaging techniques like CBCT. However, due to ethical and clinical considerations—including radiation exposure, the anatomical location of the defects, and routine clinical practices—we chose not to use CBCT, in accordance with the ALARA (as low as reasonably achievable) principles. This decision may limit the resolution of our structural evaluations, especially after augmentation. Additionally, the study only included a blank control group with no grafting material. While this approach mirrors common clinical practices where cystic bone defects are allowed to heal spontaneously, the absence of a control group treated with a non-coated allograft makes it challenging to determine the specific effects of the albumin coating. Future trials should include such control groups to more accurately evaluate the distinct impact of albumin. Finally, the study did not systematically analyse postoperative complications such as pain, swelling, or infection, which could affect patient outcomes and the success of the graft. Future research should include these clinical endpoints to provide a more thorough assessment of treatment effectiveness.

## 5. Conclusions

In summary, incorporating BoneAlbumin^®^ into the body promotes the formation of a bone structure that closely resembles the original tissue. It exhibits ideal characteristics in terms of density, hardness, and stiffness. This integration is associated with a narrower demarcation at the graft-host boundary, reduced postoperative pain, and simpler application methods, all of which contribute to shorter treatment durations. BoneAlbumin^®^ shows potential in reducing the risk of recurrence and effectively healing bone defects after procedures like cyst removal. Our assumption that patients treated with BoneAlbumin^®^ experience faster bone regeneration within a shorter timeframe has been identified. Future research should include larger patient groups and extend follow-up periods to gather more evidence and gain a comprehensive understanding of the outcomes.

## Figures and Tables

**Table 1:** Participants’ basic characteristics. The study group consists of cases where the lesion was left untreated after the removal of an odontogenic cyst, while the treatment group refers to cases where BoneAlbumin^®^ graft was used. A significant complication of the procedure, fistula formation, was compared between the two groups. Continuous variables were expressed as means with standard deviations. The statistical differences between the two groups were analysed using the *t*-test * and chi-squared test **. The significance levels were established at *p* < 0.05. SD = Standard deviation. The findings presented in this table are elaborated upon in greater detail within the main text.

Parameter	Study Group (*n* = 15)		Control Group (*n* = 15)		*p*-Value
Age (mean years ± SD)	41.6 ± 12.5		43.6 ± 14.37		0.37 *
Sex (men/women)	7/8		9/6		0.46 **
Complication (fistula), *n* (%)	2	(13.3%)	1	(6.6%)	0.67 **

**Table 2:** Mean and SD values of the defect sizes postoperatively and at 6- and 12-months follow-up in both the control and the BoneAlbumin^®^-graft groups. SD = Standard deviation.

	Control Group (*n* = 15)	BoneAlbumin^®^ Group (*n* = 15)
Postoperative (mean mm ± SD)	17.97 ± 4.98	18.4 ± 6.56
6-month follow-up (mean mm ± SD)	16.69 ± 4.53	18.11 ± 6.53
12-month follow-up (mean mm ± SD)	15.23 ± 3.67	17.66 ± 6.52

## Data Availability

The data are available upon request.
